# The potential impact of the demographic transition in the Senegal-Gambia region of sub-Saharan Africa on the burden of infectious disease and its potential synergies with control programmes: the case of hepatitis B

**DOI:** 10.1186/s12916-018-1100-0

**Published:** 2018-07-25

**Authors:** John R. Williams, Piero Manfredi, Alessia Melegaro

**Affiliations:** 1 Department of Infectious Disease Epidemiology, School of Public Health, Faculty of Medicine, Imperial College London, St Mary’s Campus, Norfolk Place, London, W2 1PG UK; 2 Dipartimento di Economia e Management, University of Pisa, via Ridolfi 10, 56124 Pisa, Italy; 3 Dondena Centre for Research on Social Dynamics and Public Policy and Department of Social and Political Science, Bocconi University, Via Roentgen 1, 20136 Milan, Italy

## Abstract

**Background:**

Sub-Saharan Africa (SSA) continues to suffer high communicable disease burdens as its demographic transition (DT) proceeds. Although the consequent changes in population structures influence age-specific contact patterns relevant for transmission, the age distribution of immunity, and the disease burden, investigation of the potential of DT to affect infectious disease epidemiology in regions of SSA has hitherto been overlooked. With a substantial disease burden and complex epidemiology, hepatitis B virus (HBV) represents a prime example of an infection whose epidemiology may be significantly influenced by the DT.

**Methods:**

An age-structured mathematical model for HBV in the Senegal and Gambia (SG) region was set within a demographic framework with varying vital rates mirroring the entire course of the DT there over 1850–2100, to investigate the effects of the DT on HBV epidemiology, with and without the combined action of vaccination. The model was run from its reconstructed *ancien régime* (old order) demo-epidemiologic equilibrium and calibrated against SG 1950 age-distribution estimates and Gambian pre-vaccination HBV age-prevalence data.

**Results:**

The model, which reproduced well demographic and HBV age-prevalence data, predicted a complex transition of HBV epidemiology over the course of the DT. This included a prolonged epoch of expansion alongside population growth and rejuvenation until 1990–2000, followed by a dramatic retreat, mainly reflecting projected fertility decline during the twenty-first century. This transitional pattern was mostly explained by the underlying demographically driven changes in horizontal transmission resulting from the changes in the age structure of the population. During 2000–2150 the HBV burden is predicted to decline by more than 70% even in the absence of vaccination.

**Conclusions:**

Demographic change alone may strongly affect HBV disease burden and shape HBV endemicity. The onset of the demographically driven decline in HBV prevalence, aligned with the expansion of HBV vaccination, forms a synergy potentially boosting effectiveness of control. Such a synergy currently appears to be presenting a “window of opportunity” facilitating HBV elimination which it would be important to exploit and which underlines the importance of taking demographic change into account when assessing the potential longer term impact of vaccination and other control measures.

**Electronic supplementary material:**

The online version of this article (10.1186/s12916-018-1100-0) contains supplementary material, which is available to authorized users.

## Background

Sub-Saharan Africa (SSA), the world region with the highest burden from communicable diseases [1], is currently experiencing a fertility transition [2], contributing jointly with continued mortality decline and massive urbanisation, to an overall demographic transition (DT). However, an investigation of any potential effects on the epidemiology of infectious disease in areas of SSA (or indeed in the wider world) has hitherto been largely neglected.

Although demography is continually in transition, the conventional idealised picture of DT, as experienced by industrialised countries [2–4] after 1750–1800, is of an initially stationary population characterised by high mortality and fertility rates — the *ancien* or *Malthusian régime* [3] — which first experiences falling mortality rates accompanied by rapid population growth leading to a young age distribution. This mortality decline is later followed by a fertility decline with, in this idealised scenario, the eventual attainment of a *modern* stationary regime at low levels of fertility and mortality. In classical demographic and demo-economic explanations, mortality decline has been the major trigger of fertility decline, which in turn resulted as the major engine of economic development of industrialised countries [3]. The experience of industrialised countries after 1960 has shown however that replacement fertility is by no means a necessary outcome of this process, with the gradual spread of below replacement fertility, in some cases even to very low levels [5], yielding rapid population ageing.

In the different regions of SSA, the joint action of fertility and mortality decline and rapid urbanisation — not to mention HIV/AIDS — are producing marked changes in population structures, primarily in the distribution of the population by age. These changes in the population age distributions are likely to affect the proportions of people of different age groups with whom individuals come into contact day by day. Such patterns of contact by age are key determinants [6, 7] of the patterns of transmission of infectious diseases and, consequently, of the ensuing age distribution of immunity. Moreover, severity of disease can often be influenced by age at infection [6], so that the overall population burden of disease also may change as the age distribution of the population changes. For infections that are vertically transmitted, time changes in age-specific fertility, combined with an evolving female age distribution and age distribution of female immunity, will also likely have an impact on the epidemiology of the infection [6].

The aim of this work is to provide a general investigation of the overall effects that changes in population age structure associated with DT might have upon the burdens of infectious diseases with complex epidemiology, focusing on hepatitis B virus (HBV), and selecting the region of Senegal and The Gambia as our “laboratory” in SSA. Only a few studies have so far investigated the possible effects of demographic change on infection transmission dynamics and control. Moreover, most such studies have focused on common childhood infections such as measles and varicella in view of their epidemiological characteristics, primarily a single well-identified transmission route [8–19]. The only work so far that has considered an infection with more complex epidemiology has focused on dengue [20], and that study confirmed by statistical analyses effects similar to those predicted in the aforementioned literature.

HBV infection is a major example of an infection causing a substantial continuing burden of disease worldwide [21–23], including cirrhosis and liver cancer; thus, it calls for a redoubling of efforts and prioritisation of actions aimed at elimination [22]. HBV has a complex epidemiology in which such key processes as transmission, infection, and disease development are strongly age-related [24–26]. More specifically, HBV has multiple transmission routes (see Additional file 1: Figure S3.1), with perinatal, sexual, and horizontal transmission by person-to-person contacts being most prominent in the general population. Each of these transmission routes is potentially affected by changes in the age distribution of the population. In addition, the probability, once infected, of developing persistent HBV infection is strongly age-related, being very high in infancy but declining steeply with age [25]. Given these age dependencies, it is presently unclear how the ongoing DT might affect the overall transmission and resulting disease burden of HBV. Finally, the pre-vaccination landscape of HBV exhibited a dramatic epidemiologically significant variation in prevalence worldwide [27]. In Medley et al. [28] an explanation has been proposed for this variability by the nonlinear feedback between the HBV force of infection and the age-related probability of developing HBV carriage; moreover, it was conjectured that the demographic changes along the DT might have been a major determinant of this heterogeneity.

Mathematical modelling of infectious disease transmission dynamics is a technique which naturally lends itself to the investigation of the interplay between population and infectious disease dynamics. We used an age-structured model for the transmission dynamics of HBV with realistic population dynamics parameterised with demographic (vital rates) and epidemiological data (HBV prevalence) from Senegal and The Gambia, considered here as a unique demographic entity, in an attempt to cast light on the nature and extent of the potential influence of the course of the DT on HBV epidemiology and its possible implications for the variability of HBV prevalence worldwide.

## Methods

### Senegal and The Gambia

Focus on Senegal and The Gambia as a unique demographic entity (denoted from now on as SG) was motivated by the fact that The Gambia, apart from its coastline (< 50 km), is entirely surrounded by Senegal, and also that there is a strong ethnic and cultural overlap between the two countries, which for a period in the 1980s together formed a loose confederation, Senegambia [29]. The Gambia itself is an exemplar of a country in SSA with good-quality data on the prevalence of pre-vaccination HBV infection by age [25]. Moreover, the two countries supplied a substantial portion of the data used previously to estimate a key function relating age and likelihood of becoming a carrier [25]. In addition, although patterns of age-specific growth rates of the small population in The Gambia are somewhat erratic, this is not the case for the much larger Senegal, which has exhibited, according to United Nations (UN) data, fairly regular patterns of DT and mortality decline since 1950, and a well-separated late onset of fertility transition (by about 1990).

### Data

Data on previous experience of HBV infection by age were drawn from published work on The Gambia [30]. Plausible ranges for HBV epidemiological parameters relating to infectivity, duration of stages of infection, etc. (Additional file 1: Table S2.1) were gleaned from the literature [23]. Demographic data on fertility and mortality rates in Senegal and The Gambia were drawn from the UN 1950–2015 estimates and the subsequent 2015–2150 projections, as well as data on the population age distribution by 5-year age groups which were used for model validation [31]. In particular, data from all three main UN projections variants, namely, the “medium” one, which is the UN key variant taken as our baseline here, as well as the “high” and “low” variants, were considered. Moving averages of estimated or projected UN age-specific fertility and mortality rates in successive 5-year periods from 1950 to 1955 to 2095–2100 provided time-varying vital rates for the model over a 150-year period.

### The mathematical model for population and HBV transmission/disease dynamics

**Fig. 1:**
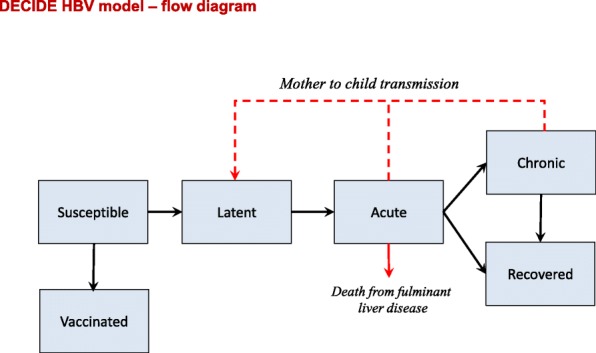
Model of HBV transmission. Flow diagram showing HBV model compartments and flows between them (*black arrows*); *dashed red line* represents vertical transmission from mothers with acute or chronic infection to their newborn children

The population model was age-sex structured with time-changing age-specific fertility and age- and gender-specific mortality rates to depict a realistic time course for the DT. The part of the model simulating the transmission dynamics of HBV (see the flow diagram in Fig. 1) included age dependencies in vertical, horizontal, and heterosexual modes of transmission, as well as in the probability of developing HBV carriage once infected (see Additional file 1: Figure S3.1 and Text S2 for details including the full partial differential equations (PDEs) describing the model). A computer program designed for the purpose and coded using Fortran 77 provided for numerical solution of the model PDEs.

In the model all births to acutely and chronically HBV infected mothers were at risk of vertically transmitted HBV, acquired at a rate depending on the relative proportions of females with acute or persistent (i.e. chronic) infection in each fertile age group. A proportion of vertically infected offspring was assumed to develop chronic infection [25]. Children and adults at all ages were at risk of horizontal HBV transmission at an age-specific rate determined by an appropriately specified WAIFW (who acquires infection from whom) matrix [6] obtained by multiplying the WAIFW matrix estimated for The Gambia by Edmunds and colleagues [30] by a single scaling parameter for purposes of data fit. The influence of the evolving age distribution of the population on social contact patterns relevant for horizontal HBV transmission, and therefore on the WAIFW, was modelled in accord with previous work [10–12, 16]. Sexual transmission started at the age of sexual debut, assumed to be 15 years, at a rate dependent on (1) the rate of acquisition of new partners for each gender and age group, (2) sexual mixing matrices determining the proportion of sexual contacts to be drawn from each age group, (3) the proportion of acutely and chronically infected partners in each gender age group, and (4) the risk of transmission per acutely and chronically infected partner. The sexual mixing matrix adopted obeyed a preferred mixing rule [32] with a parameter determining the degree of age assortativeness of sexual partnerships. The underlying age-gender specific rates of sexual behaviour are kept constant during population evolution, but as male and female populations evolve separately, at each time point an adjustment is made to partner acquisition rates to ensure that male and female partnerships always balance. Note that the preferred mixing rule, in which the force of infection acting on each age-gender group explicitly depends on the proportions of the population in the various age groups, straightforwardly incorporates the feedback of the evolving age distribution of the population on sexual contact patterns.

Upon infection by any route of transmission, susceptible people moved to a latently infected but noninfectious stage and subsequently to an acutely infected and infectious stage, a small proportion of the latter being assumed to die as a result of fulminant liver disease. Following acute infection, a strongly age-dependent proportion [25] moved to a state of chronic carriage having an average duration of several decades prior to recovery, while the complementary proportion resolved the infection, moving directly into the recovered stage; for simplicity, it was assumed that there was no additional disease-specific mortality during chronic carriage and that no screening and treatment programme was in place which might hasten recovery or reduce infectivity.

The model could also be set to include HBV vaccination at birth and/or at any subsequent age point, starting from a pre-specified time point, with a vaccine assumed to have 100% efficacy and lifelong duration. Those vaccinated in the model are moved to a vaccinated and immune compartment.

### Parameterisation of the demographic component

**Fig. 2:**
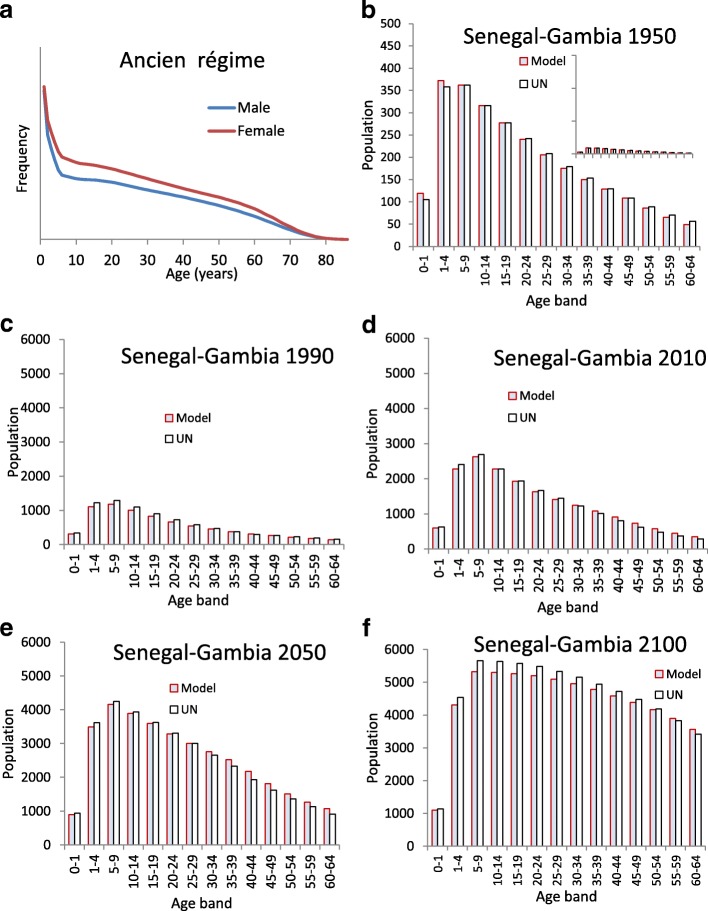
Results from the demographic component of the model for the SG population. **a** Male and female age profiles of the SG population at *ancien régime* equilibrium corresponding to the years 1840–1850 in the model. **b**–**f** Model predictions for the age distribution by 5-year age groups up to age 65 of the SG population at specific time points compared with UN estimates 1950–2100 (note that inset in **b** has same vertical scale as **c-f**)

Estimated (period 1950–2015) and projected (2015–2100) mortality and fertility rates from the UN for Senegal and Gambia were pooled to obtain a unique set of rates for SG as a single entity. In order to depict the course of an entire DT for SG, from its *ancien régime* stationary demographic equilibrium, we hypothesised that SG fertility at 1950 reflected true SG fertility through its entire previous history back to when the *ancien*-demographic *régime* prevailed. We then projected mortality rates into the past to reconstruct a reasonable *ancien régime* mortality table and the related stationary population (as expected for the *ancien régime*), i.e. one characterised by a constant total population size and an invariant age distribution, (see Additional file 1: Text S1 for details). The stationarity of the *ancien régime* age distribution was ensured by requiring that the corresponding female life table once combined with SG 1950 female fertility rates promoted a net reproductive rate of the population equal to one. This equilibrium population was taken as the initial condition for the age distribution of the SG population, i.e. the one prevailing before destabilisation due to the onset of the mortality transition (Fig. 2a). Departing from the *ancien régime* equilibrium, the demographic model was then run forward in time to reproduce the DT in SG using the pooled set of SG mortality and fertility rates. Model predictions were compared, by a least squares criterion, to the 1950 SG male-female age distribution, assuming smooth adjustment over time of *ancien régime* mortality to the 1950 UN estimate.

### Parameterisation of the HBV component of the model

We assumed also that HBV was, in the demographic *ancien régime* equilibrium, at an epidemiologic equilibrium which itself was destabilised by mortality decline at onset of the DT. Based on the HBV literature, uncertainty ranges were specified for HBV epidemiological parameters in the model, and Latin hypercube sampling (LHS) was used to explore these parameter ranges (see Additional file 1: Table S2.1 for full details of HBV parameters included in the LHS procedure). By LHS we generated 24,000 sampled parameter sets. Departing from the *ancien régime* demo-epidemiologic equilibrium, the full HBV model was first run forward for each sampled parameter set until 1984 using smoothed UN-based vital rate estimates 1950–1985 [31], under the hypothesis that no interventions (vaccination, use of protection for sexual activity, etc.) were in place. Model predictions at 1984 were compared, by a least squares criterion, to Gambian early 1980s’ HBV pre-vaccination age-prevalence data on both past exposure and current infection. As best fit we took the parameter combination generating the lowest value of the least squares function over the entire set of 24,000 LHS samples. In particular, the horizontal transmission WAIFW matrix was fitted to the data by adjusting the scaling factor applied to all the elements of the matrix within the overall fitting procedure. During each simulation the HBV parameters were kept constant over time, ensuring that any changes over time in results for disease prevalence arose solely from demographic change. Uncertainty bands about the best-fit set of parameters were generated by retaining the 2400 (i.e. 10% of the total) parameter sets generating the largest values of the least squares function. These uncertainty bands about parameter values were then used to generate uncertainty bands about the predicted HBV trajectories over time.

### Parameterisation of HBV vaccination

Our baseline vaccination scenario was designed to approximate the Senegal situation in view of its possible larger relevance as: (1) The Gambia could be considered a special case due to the work of The Gambia Hepatitis Intervention Study [33], (2) the Senegal population is many times the size of its neighbour, (3) in common with many other countries in SSA, in Senegal the lack of availability of monovalent HBV vaccine prevented the implementation of universal vaccination at birth [34]. Thus, in our baseline scenario, vaccination is administered to children aged 3.5 months, with effective coverage of 46% [34] and 2005 as year of introduction of the vaccine into the Expanded Programme on Immunisation [34]. Alternative scenarios were considered including higher coverage levels (55% and 75%) as well as the possibility of vaccination at birth to prevent vertical transmission.

### Prediction of the course of the demographic transition in SG

As a preliminary step, using the best demographic model, predictions for the age-sex distribution of the population for 1955–2100 were systematically compared with the corresponding UN estimates/projections, in order to ensure that the model-based prediction of the evolution of the age-sex structure depicted a course of the DT in SG always close to UN projected figures.

### Prediction of the long-term transitional epidemiology of HBV

The model was then used to represent the evolving epidemiology and burden of HBV (with and without vaccination) during the entire course of the DT departing from its *ancien régime* pre-transitional epidemiological equilibrium and continuing until 2150. Extension of the horizon to 2150 (rather than 2100, which is the last year of UN projections) was carried out by freezing mortality and fertility rates for 2100–2150 at the levels projected for 2100. This was motivated by the need to explore the ultimate consequences of the demographic changes projected up to 2100, which will continue to develop for several decades further despite demographic rates having been kept constant thereafter. For the future evolution of HBV we focused — as baseline — on the UN “medium” variant, but sensitivity analyses considering the implications of the other main UN variants, namely the “high” and the “low” ones, were also undertaken, as well as a worst-case scenario where fertility is maintained constant at its maximal level (achieved during 1990–1995). This also allowed consideration of the potential long-term effects on HBV epidemiology in SSA of the eventual fall into continued below replacement fertility.

## Results

### Fit and reproduction of demographic and HBV data

**Fig. 3:**
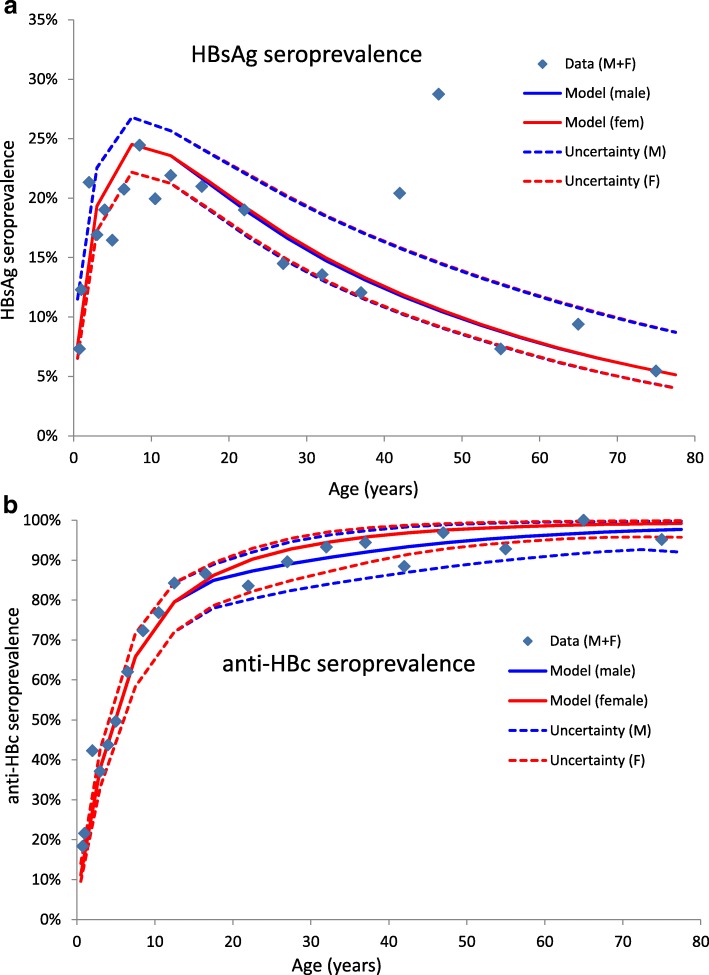
Best model fit to HBV prevalence. **a** Model-predicted age-specific prevalence of exposure to HBV (represented by HBV core antibody prevalence) compared with prevalence observed in The Gambia in the early 1980s [30]. **b** Model-predicted age-specific prevalence of serological markers of persistent infection (corresponding to HBV surface antibody) compared with corresponding prevalence observed in The Gambia in 1984 [30]. Results show best-fit model results and related uncertainty bands based on those parameter constellations from LHS representing the best 10% values obtained for the least squares score function

The model identified 1840–1850 as the epoch when the *ancien regíme* equilibrium ended due to initiation of the mortality transition (Fig. 2a). Forward projections of the demographic model satisfactorily fitted the 1950 UN estimate of population age distribution for both sexes (Fig. 2b). Subsequent model-based forward projections of the age-sex distribution remained quite close to UN population estimates and projections for the entire horizon 1950–2100, as reported in Fig. 2b–f for the case of the UN “medium” projection variant. The model also reproduced quite satisfactorily the 1980s’ pre-vaccination age-prevalence data for exposure to HBV (Fig. 3a) and current HBV infection (Fig. 3b).

### Effects of the demographic transition on HBV epidemiology in the absence of vaccination

**Fig. 4:**
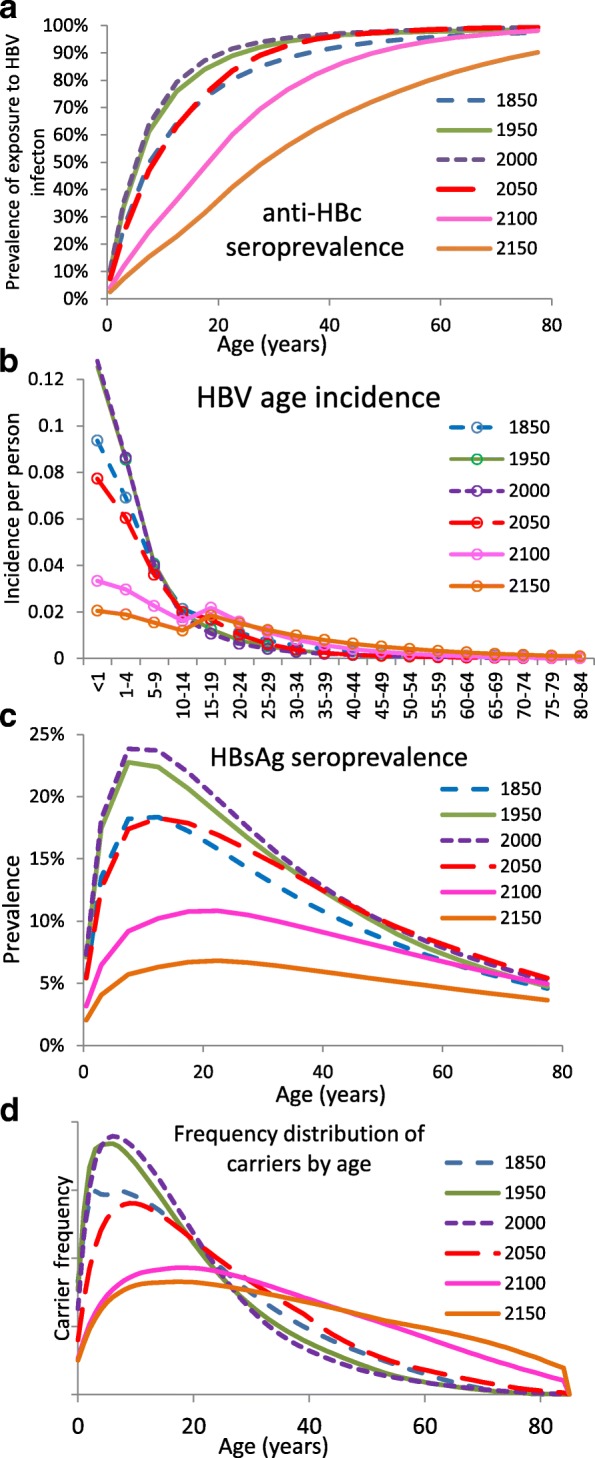
Model results for temporal change in HBV prevalence and incidence under UN medium variant. Predicted evolution of HBV epidemiology during the entire course of the DT at specific time points. **a** Age-specific prevalence of exposure to HBV infection; **b** age-specific HBV incidence; **c** age-specific prevalence of chronic HBV infection; **d** frequency distribution of cases of chronic infection by age (computed as the ratio between carriers at each age and the total number of carriers in the population)

**Fig. 5:**
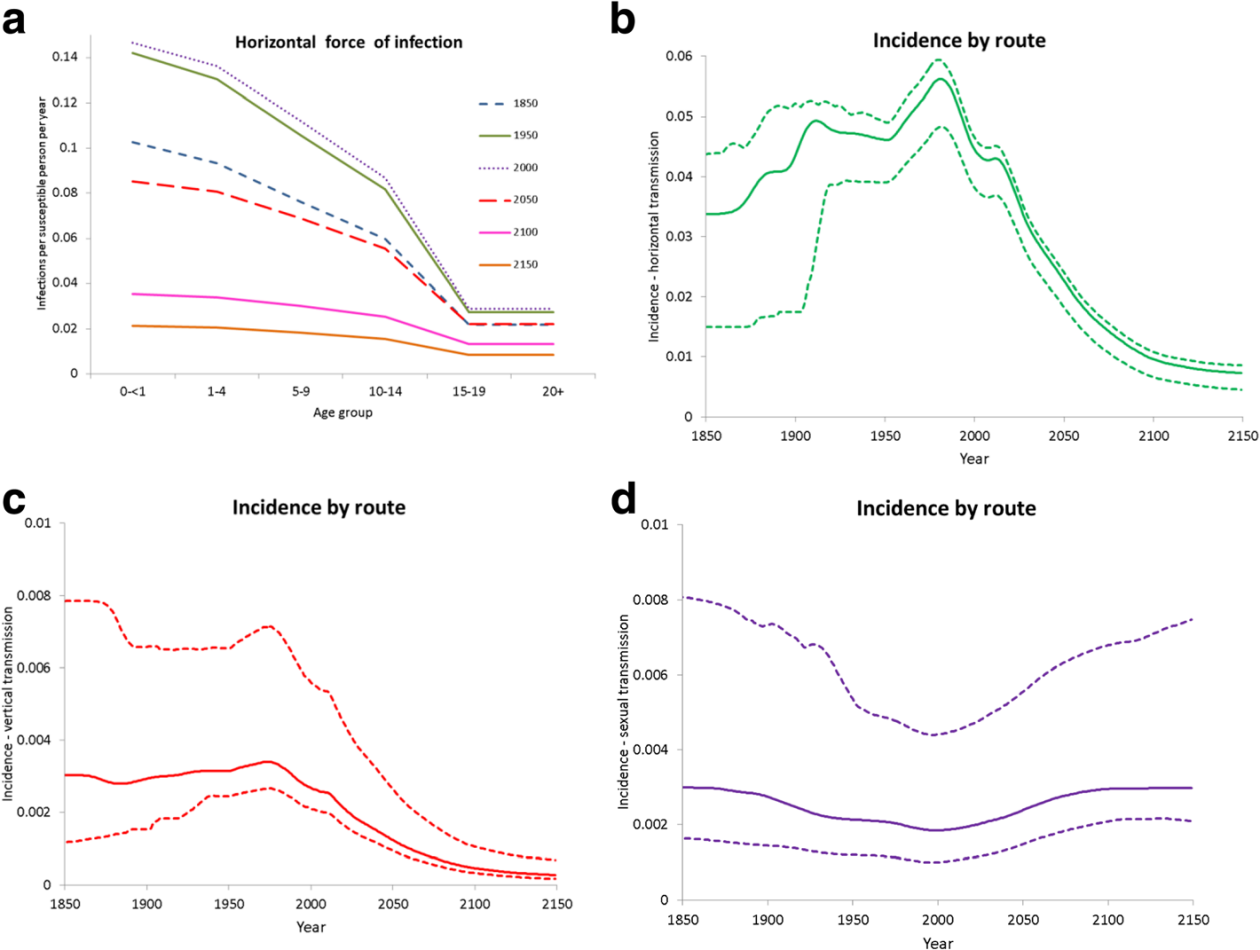
Model results for temporal change in horizontal force of infection and relative importance of transmission routes under UN medium variant**. a** Predicted evolution of the age-specific HBV force of infection from the horizontal transmission route at specific time points over the entire course of the DT. Predicted HBV incidence by transmission route over the entire course of the DT: **b** horizontal, **c** vertical, and **d** sexual (Note the order of magnitude difference in vertical scales for horizontal transmission (**b**) and vertical and sexual transmission (**c**) and (**d**))

Figures 4 and 5 report predictions by the best model for the evolution of the natural history of HBV that would be observed in the absence of immunisation during the whole course of the DT in SG, from around 1850 (corresponding to the *ancien régime*) to 2100 according to the UN medium variant. As well summarised by the transitional trend of prevalence of exposure to HBV infection (Fig. 4a), starting from the HBV pre-transitional equilibrium profile at 1850, where prevalence by age 15 was in the region of 70%, HBV was predicted to gradually expand, reaching prevalence levels as high as 85% by age 15. Thereafter HBV entered a phase of continuing decline, returning to levels comparable to, and subsequently below, the pre-transition phase when projected fertility approached and stabilised near to replacement levels (between 2100 and 2150). At 2150, HBV prevalence (in the absence of any control measures) is predicted to settle at 25–30% by age 15. This same story is told by the other graphs of Fig. 4: in particular, the population age distribution of disease burden post-equilibrium (Fig. 4d) remained heavily skewed towards younger ages until 1950–2000 before becoming substantially more evenly distributed in later years, reflecting the changed demography.

Much of the explanation for the predicted trends lies in the dramatic changes of horizontal transmission during the DT. This is made clear by the temporal trend of the corresponding age-specific force of infection (Fig. 5a) among young individuals (aged below 15 years). Indeed, the horizontal force of infection among the youngest systematically increased during the HBV expansion phase up to levels of around 15% per year, approaching levels commonly seen for typical childhood infections such as varicella [35]. This resulted from the continued rejuvenation of the population during the major epoch of mortality decline among young individuals and persistent high fertility (roughly up to 2000). Subsequently, the horizontal force of infection among young individuals experienced continued decline, falling to very low levels — well below those of the *ancien régime* – during the major phase of fertility decline (after 2050) and corresponding progressive population ageing. This pattern arises as a consequence of the dramatic changes in the proportions of social contacts between younger and older individuals during the various stages of the DT [11, 12, 16, 17]. Notably, vertical and sexual transmission routes also showed complex patterns of evolution during the course of the DT, as signalled by the changes in route-specific incidences (Fig. 5b–d), even under the assumption of constant sexual behaviour rates. In the *ancien régime*, horizontal vs sexual transmission accounted for 85% and 8% of overall HBV incidence, respectively. During the HBV expansion phase, the two routes are predicted to diverge, with horizontal incidence growing to a maximum in the 1980s and sexual incidence declining to a minimum not long after the 1990s, when the horizontal route is predicted to account for almost 92% of total incidence. In the 1990s, after horizontal transmission has begun its long decline, sexual transmission inverts its trend, returning to *ancien régime* levels by around 2060; this pattern of incidence due to horizontal transmission is mirrored, at an order of magnitude lower, by that for vertical transmission, results well reflecting observations from The Gambia [36].

### Impact of vaccination on HBV burden under different demographic transition scenarios

**Fig. 6:**
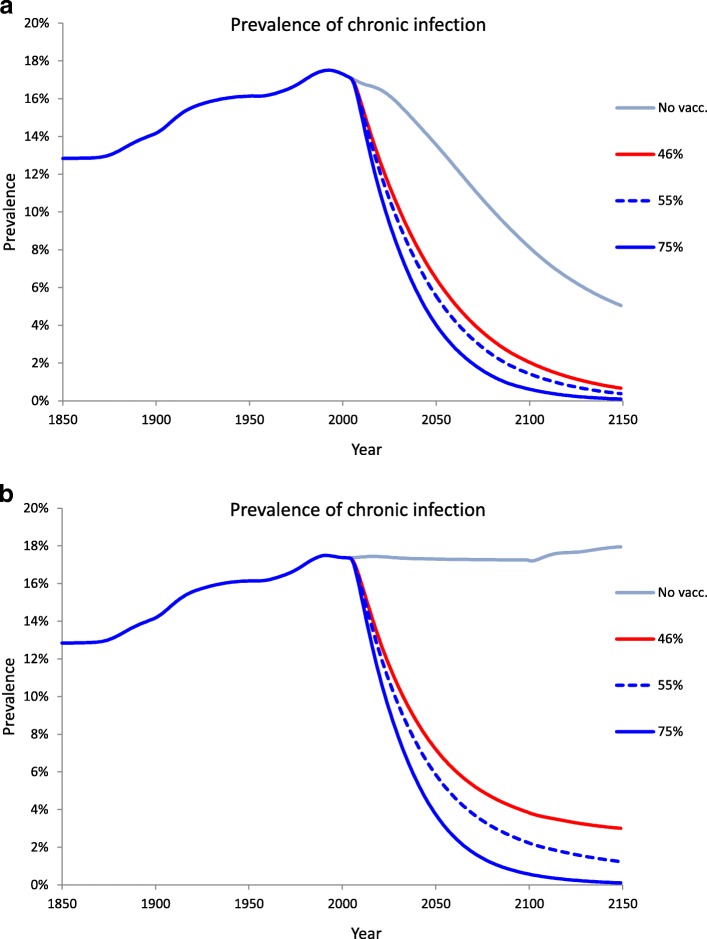
Potential synergy between demographic transition and HBV vaccination under different projection variants. Reconstructed and projected overall population burden of HBV disease (over the entire course of the DT up to 2150) without and with vaccination commencing in 2005: **a** the case of UN medium variant; **b** a worst-case scenario where fertility remains constant at its maximum level. Vaccination is administered at age 3.5 months assuming 100% efficacy

To separate the effects of the continuation of the DT from those of vaccination, we focus on the proportion of the overall population with persistent infection, which serves as a useful summary measure of burden of disease. Under the best model in the absence of any immunisation, this increased monotonically along the DT (in the medium variant scenario) to a peak of 17% in ~ 1992, i.e. a decade prior to the introduction of universal HBV vaccination in Senegal in 2005, before decreasing steadily thereafter (Fig. 6a, uppermost line), reaching levels as low as 5% in 2150.

Figure 6 also investigates the effects of possible vaccination programmes (all initiated in 2005) comparing our baseline DT scenario in SG (Fig. 6a) with the alternative scenario where SG total fertility is frozen at its maximal level observed in 1990–1995, namely before the fertility transition initiated (Fig. 6b), the scenario representing the worst case in terms of infection control given that it completely prevents the phase of HBV retreat. The differences are stark. If fertility remains constant at its highest level, the prevalence of chronic HBV in the absence of immunisation would remain nearly constant over time at its maximum level (Fig. 6b, uppermost line). At baseline coverage (46%), chronic HBV would cease its decline at a level of around 3% prevalence, whereas coverage in excess of 75% would be effective in bringing the infection under full control.

In contrast, the onset and completion of the DT (along the medium scenario) is predicted to “sustain” immunisation by yielding a substantial decline in the prevalence of chronic HBV (down to 1% by 2150) even with the baseline coverage, and bringing it to near elimination level with a coverage just in excess of 55%. This suggests the possibility of a strong synergy between vaccination and the HBV retreat phase triggered by the DT. The impact of a hypothetical alternative programme of vaccination at birth at the same rate of 46% shows an even steeper decrease in the prevalence of chronic infection (see Additional file 1: Text S4).

**Fig. 7:**
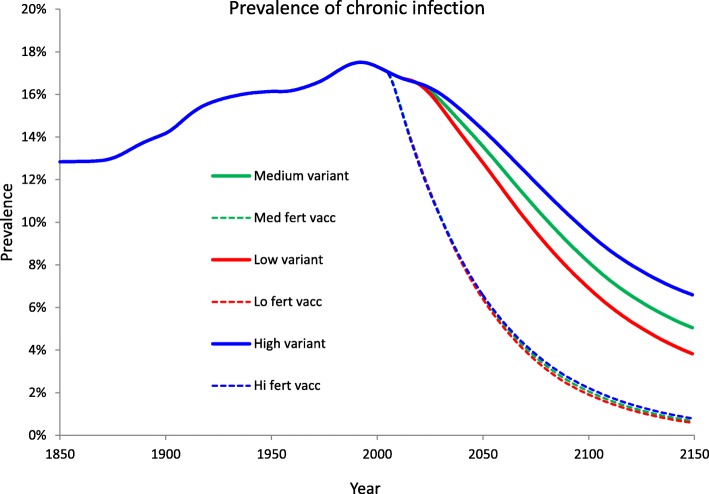
Influence of UN projection variants on the projected burden of disease. Results for the overall prevalence of chronic HBV infection under scenarios of no vaccination and baseline vaccination (coverage of 46% at 3.5 months of age) corresponding to the high, medium, and low fertility UN projection variants 2015–2150

Finally, the impact of different patterns of DT in the future, as embedded in the “low” and “high” variants of UN projections (in contrast to the “medium” one), and the interplay of this with vaccination are also reported (Fig. 7). The persistent fall into below replacement fertility, as hypothesised in the low variant, would made control conditions easier but only after 2050.

## Discussion

Demographic change can, according to circumstances, create great challenges and opportunities. Possibly the major example in history is represented by the interplay between DT and the industrial revolution, with the acknowledged role of mortality decline as the major trigger of fertility decline, via the switch from quantity to “quality” of children, which in turn resulted as a major engine of the sustained economic development of industrialised countries [3]. Nonetheless, it has remained largely unclear where the balance may lie in terms of its effect on infectious disease epidemiology. As age distributions of infection and immunity are far from uniform, patterns of contact between different age groups are of fundamental importance for infection transmission, so that change in population age structure may strongly influence infectious disease epidemiology. It then becomes essential to attempt to understand how the two interact, especially for those world regions, such as SSA, where the burden from infectious diseases is still striking.

In SSA, although mortality decline possibly had its beginnings prior to 1900 [3], much of the process has taken place since 1950 and is still ongoing. On the other hand, the fertility transition was unexpectedly delayed and has been proceeding at a slower rate compared with other regions such as Asia and Latin America [2]. Indeed, fertility decline began, in regions such as SG, as recently as the 1990s [2] and is predicted to continue to display its effects over a span of a century or so. The resulting massive changes in the age distribution of the populations are likely to have greatest impact on those infections in which processes of transmission and disease are strongly age-related and infection may be long-lasting. HBV is one such infection and one which gives rise to a substantial burden of disease. Global mortality associated with viral hepatitis ranks equally with that from HIV/AIDS and tuberculosis, and in 2016 the World Health Assembly adopted a strategy for its global elimination [21], with HBV being responsible for the majority of deaths associated with viral hepatitis [22]. Nonetheless, the great heterogeneity in the global distribution of the burden of disease arising from HBV [27, 28] might hinder steps towards its elimination. For these reasons a mathematical modelling approach may provide a key to disentangling the mutual interplay of demographic changes and HBV epidemiology during the different phases of DT, and, by depicting an entire range of dynamic regimes, possibly offer some clues towards explaining the puzzle of the heterogeneity in HBV burden.

Using a mathematical model, we assessed the possible extent of the impact of the entire course of the DT on the transmission and burden of HBV in a region of SSA. Our results predict that, departing from the pre-transitional demo-epidemiologic equilibrium prevailing at some time prior to 1900 and later destabilised by the onset of the mortality transition, HBV burden may have followed a complex “epidemiological transition” pattern. This pattern is initially characterised by a long epoch of expansion until ~ 2000, corresponding to the epoch of maximal population rejuvenation, and it is predicted to be subsequently followed by a dramatic “retreat” corresponding to the major epoch of fertility decline observed from 1990 onward and continuing for decades after the completion of fertility decline. This demographically driven pattern of expansion-retreat of HBV is mostly explained by the underlying dramatic changes in horizontal transmission due to the changes in the age distribution of the population. During the HBV expansion phase, this change in transmission is the consequence of the period of rejuvenation following mortality decline in young age groups while fertility remained persistently high during the first stages of the transition. During the phase of HBV retreat, the change in transmission is mostly the consequence of the decline in fertility associated with the onset and completion of the fertility transition in SG. Thereafter, between 2000 and 2150 (when below replacement fertility prevails), HBV burden is predicted to decline dramatically by around 70%.

These results seem to be of importance from a number of standpoints. First, they shed light on the nature, extent, and determinants of the potential influence of demographic change on the burden of HBV, and quite possibly other infections with a similarly complex epidemiology having in common age dependency in key epidemiological processes. Second, they supply a contribution to the interpretation of the long-term natural history of HBV epidemiology. Third, they provide considerable evidence in support of a conjecture formulated in [28], namely that the great variability in pre-vaccination HBV prevalence worldwide might largely be a consequence of the DT. More precisely, the present work has shown that the greatest part of this variability might result from the differences in the timing of onset and pace at which the DT was experienced in different regions of the world [2, 3]. Fourth, they indicate a precise causative role of the DT in triggering “epidemiological transitions”, broadly interpreted as patterns of retreat of the burdens of disease which are a key component of Omran’s classical definition [37]. Last, the onset of a demographically driven decline in HBV prevalence in the model coincided with a real-world expansion of HBV vaccination, suggesting a synergy potentially boosting effectiveness of control in the decades leading up to elimination. This appears to be of fundamental importance in helping to achieve the aim of HBV global elimination [21]. Indeed, the predicted initiation of the HBV retreat phase in the last 15 years suggests that a “window of opportunity” has presented itself where fertility decline will synergistically concur with immunisation programmes to hasten the global target of HBV elimination.

Finally, although due to paucity of data our analyses focused on our characterisation of a single region of SSA, our model representations of the DT and of HBV epidemiology are fairly general. We therefore feel that a major strength of this work is that it provides a pointer for interpreting past and future long-term trends of HBV in SSA and also at the global scale.

The major limitations in studies of the impact of population change on infection transmission typically lie in the lack of availability of sufficiently long-term time series of reliable epidemiological data allowing robust estimation of the impact of demographic processes on age-related epidemiology. As here, most available studies [12, 15, 16, 18] have in common the same drawback: they have to postulate a direct influence of age-structured demographic change on transmission and typically rely on a single age-structured infection datum (e.g. a single serological profile) to estimate transmission. However, as argued in [11], the formulation of horizontal transmission adopted here, though simple, has the advantage of potentially capturing a whole range of processes triggered by fertility decline, namely the ageing of population in the groups most involved with horizontal transmission and also the ensuing decline of family size as an engine of the decline in intra-family horizontal transmission. In this study we modelled horizontal transmission by a WAIFW matrix as previously available for the study setting [30], being obliged to do so given that direct data on social contact patterns in SSA are so far available only for Eastern Africa [38], where socio-cultural patterns are believed to be markedly different from those of Western Africa and the setting considered. The lack of direct contact data in most of Africa remains a serious limitation. Another limitation lies in the lack of precise predictions about mortality resulting from chronic HBV infection, a choice motivated by a lack of appropriate data.

To sum up, while the focus of this work has been on isolating and highlighting the impact of changing demography on the epidemiology of HBV, it is important to note that patterns of evolution of social and sexual behaviour over time will add a further layer of complexity to the resulting pattern of HBV epidemiology. More generally, it is reasonable to conclude that where processes of infection and disease or relevant behaviours display age dependencies, potential changes in age distribution associated with fertility, mortality, or migration may have a significant bearing on observed patterns of infection and should be taken into account when forward planning interventions.

## Conclusions

For infections where processes of transmission, infection (including contact patterns relevant for transmission), and disease operate in an age-dependent way, demographic change can, of itself, give rise to changes in the burden of infectious disease even in the absence of changes in the processes of transmission and infection. Thus, it is clearly of importance to take account of demographic change when assessing the potential longer term impact of vaccination and other control measures focused on specific age groups.

## Additional file

Additional file 1: Model parameterisation and equations, and additional figures. (PDF 1431 kb)

## Abbreviations

DT: Demographic transition
HBV: Hepatitis B virus
LHS: Latin hypercube sampling
PDE: Partial differential equation
SG: Senegal-Gambia
SSA: Sub-Saharan Africa
WAIFW: Who acquires infection from whom
