# Supplementary material for: The potential impact of the demographic transition in the Senegal-Gambia region of sub-Saharan Africa on the burden of infectious disease and its potential synergies with control programmes: the case of hepatitis B
Source: BMC Med. 2018 Jul 25;16:118. doi: 10.1186/s12916-018-1100-0 (PMC6058373; doi:10.1186/s12916-018-1100-0)
Supplement: Supplementary file 1 — Model parameterisation and equations, and additional figures. (PDF 1431 kb) [file 12916_2018_1100_MOESM1_ESM.pdf]

SUPPLEMENTARY MATERIAL

Text S1: Parameterization of Senegal-Gambia (SG) demographics

Parameterization of our demographic model for Senegal-Gambia relies on UN data as available from the last UN projections round (UN 2015), and combines UN estimates covering the period 1950-2015 with UN projections covering period 2015-2100. For projections of future population trends in SG we used as a baseline the UN “medium” variant, but also the “low” and “high” variants have been considered in the analysis.
Age-specific mortality and fertility for SG over 1950-2100 were computed by suitably averaging the corresponding Senegal and Gambia data. Moreover, since we wanted to initialize the HBV model from a fully stationary demo-epidemiologic initial regime, and considering that already at 1950 both countries were far from stationarity in view of their large positive growth rate, resulting from the high fertility and the declining mortality, we also supplied a simple reconstruction of the mortality schedule prevailing in the SG pre-transitional demographic regime. In what follows we shall also refer to the pre-transitional demographic regime, i.e., the regime prevailing before the initiation of any mortality decline, which is typically considered the first phase of the demographic transition, as the demographic *ancien-régime*.

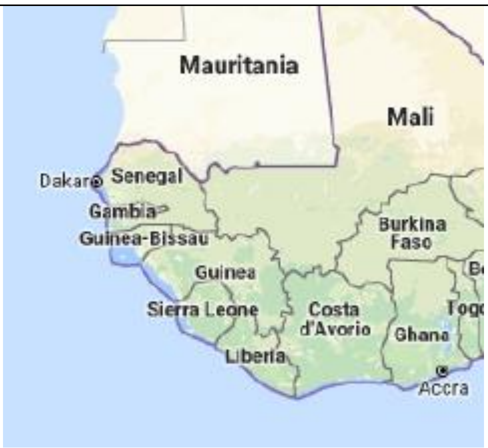

SG 1950-2100 fertility

Age-specific female fertility rates $f_t^{SG}(x,n)$ for SG for 5-years age groups (x,x+n), n=5, and for each quinquennial period (t) and during 1950-2100 were straightforwardly computed by taking the weighted average of age-specific female fertility rates (estimated or projected) for Gambia (G) and Senegal (S), using as weights the age-specific weights of female population (estimated or projected) during the same period. This follows from the simple relation:

$$f_t^{SG}(x,n) = \frac{B_t^{SG}(x,n)}{F_t^{SG}(x,n)} = f_t^{S}(x,n) \cdot \frac{F_t^{S}(x,n)}{F_t^{SG}(x,n)} + f^{G}(x,n) \cdot \left(1 - \frac{F_t^{S}(x,n)}{F_t^{SG}(x,n)}\right) \qquad (1)$$

where F_t^i (i=S,G,SG) represents the corresponding average female population during the same time period in the same age group in Senegal, in The Gambia, and in SG respectively.

The overall (estimated and projected) trend of the TFR for SG over 1950-2100 based on the underlying UN estimates 1950-2015 and the “medium” projection variant for 2015-2100 is displayed in Fig. S1.1.

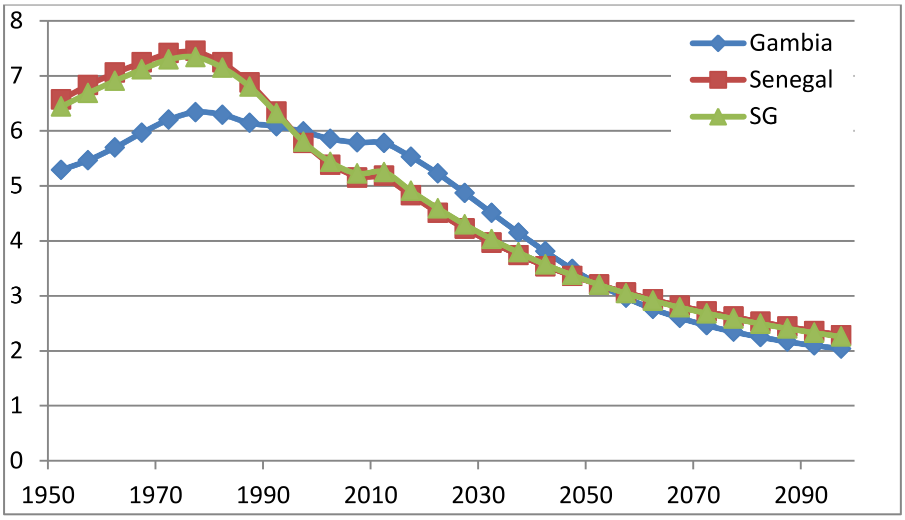

Fig. S1.1. Senegal, Gambia, and SG. Estimated + projected (according to UN “medium” variant) trend in TFR 1950-2100.

SG 1950-2100 mortality

Similarly, life tables for both sexes for SG were generated for each quinquennial period during 1950-2100, by averaging the underlying life tables for Senegal and Gambia. This was done by retaining the estimated (or projected) life tables for Gambia and Senegal, and then averaging them using the total number of deaths of observed in each country in the same period.

Reconstruction of SG demographic “*ancien-régime* “

Over period 1950-2015 Senegal and Gambia have been characterized by rapid population growth fueled by declining mortality in presence of persistently high fertility. Mortality decline surely initiated prior to 1950 but unfortunately, to the best of our knowledge, this early phase is poorly documented. In particular, the total fertility rate (TFR) increased in Senegal (Gambia) from about 6.5 (5.5) in 1950 to 7.5 (6.5) in 1980 before initiating its decline (Fig S1.1). This state of affairs implied that since 1950 onward the Senegal and The Gambia (total) population has been increasing at a large growth rate, in the region of 2.5-3.0 % per year (Fig S1.2). In particular, in the case of Senegal (which represents the large majority of the SG population), the growth rate has been fairly constant. Assuming negligible perturbation by migration this suggests that its population experienced a phase of approximately *stable* growth (Keyfitz and Caswell 2008), where a combination of persistent high fertility and not-too-fast declining mortality were promoting a coarsely time invariant age distribution. This is also documented by the substantial stability of the underlying age specific growth rates (not reported) which represent the most reliable indicators of the presence of stable growth (Preston et al 2000). The corresponding trends for the Gambia are much more erratic, possibly due to the very small population size which possibly made it very sensitive to migrations, but still the effects of the gap between high fertility and declining mortality are very clear, with an average growth rate about 3.5% during 1950-2015.

An implication of this trend is that already in 1950 Senegal and Gambia populations were far from the ideal state of *ancien-régime* stationary equilibrium[1] that we expect to have been broadly prevailing at the beginning of the demographic transition i.e., before the destabilization that occurred when mortality decline, along the *mortality transition*, initiated. Since our main hypotheses here are that also HBV was in stationary equilibrium during the demographic *ancien-régime*, and that the early mortality transition had the potential to perturb the equilibrium of HBV, in order to initialize the model from a condition of full demo-epidemiologic stationarity, we supplied a coarse reconstruction of SG demographic *ancien-régime*.

The concept of a stationary demographic equilibrium implies a stationary (over time) mortality regime (i.e., a stationary *life table*) combined with a stationary reproduction, where the typical female individual produces on average one female offspring during her entire fertile period given prevailing mortality conditions. The latter condition is expressed by requiring that the (demographic) net reproductive rate (NRR) is equal to one, where $NRR = (\frac{1}{l_0}) \sum_i \; f(x_i, n) L(x_i, n)$ where $L(x_i, n)$ denotes the number of years lived in age class $(x_i, x_i + n)$ in the relevant life table, l_0 the number of women alive at age 0 in the life table, and $f(x_i, n)$ the age-specific fertility rate in age group $(x_i, x_i + n)$.

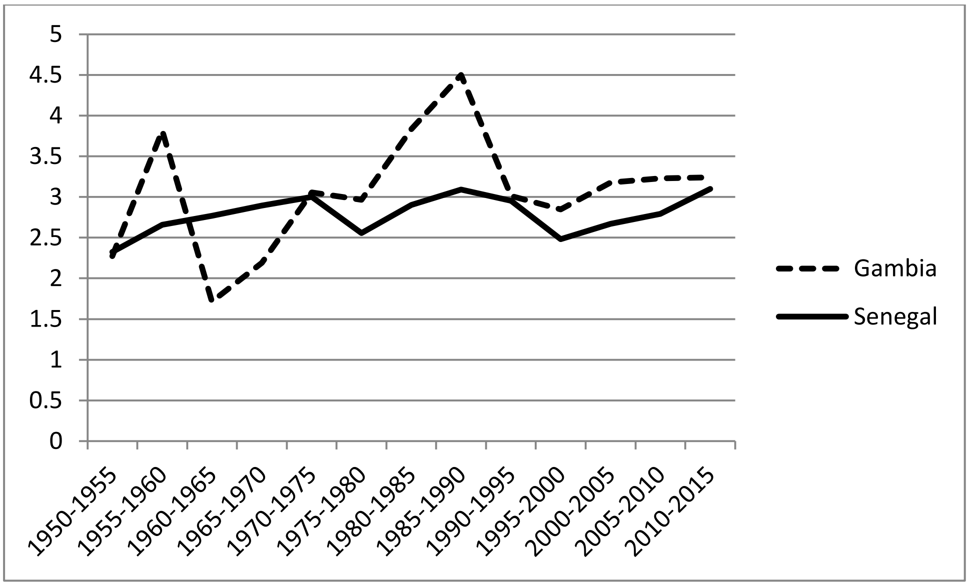

Fig. S1.2. Senegal and Gambia 1950-2015. Average exponential growth (%per year) per quinquennium. Source UN.

As a preliminary step towards the reconstruction of the *ancien-régime* mortality we made the simplifying hypothesis that our estimate of SG fertility in 1950 based on UN 1950 estimates was representative of the true SG *ancien-régime* fertility. As previously pointed out, according to UN data both Senegal and Gambia exhibited a marked increase in the total fertility rate which increased in

[1] In demographic jargon a *stationary* population is one characterized by time-invariant total size and age distribution.

Senegal (Gambia) from about 6.5 (5.5) in 1950 to 7.5 (6.5) in 1980 before plateauing and initiating to decline. Such initial phase of increase in TFR parallel to mortality decline is a well-documented fact of the fertility transition in the developing world (e.g., Dyson and Murphy 1985). Note also that the slope of the trend was already markedly positive (especially in Senegal) at 1950. This therefore suggests that some increase in fertility was likely already in place prior to 1950. Therefore, our hypothesis is to be considered as a departure point representing a useful baseline. Nonetheless the results reported in the manuscript are not sensitive to small departures from this baseline. The resulting *ancien-régime* age-specific fertility schedule $f_{AR}^{SG}(x_i, n)$ for SG is reported below in Table S1.1.

Age groups (5 yr)	15-19	20-24	25-29	30-34	35-39	40-44	45-49	**TFR**
$f_{AR}^{SG}(x_i, n)$	186.8	284.3	297.2	244.1	169.9	73.3	32.0	6.4

Table S1.1 *Ancien-régime* age-specific fertility schedule for SG

SG ancien-regime mortality

As for mortality, according to the UN, West Africa countries were characterized by a large heterogeneity in mortality profiles in 1950, with some countries e.g., Sierra Leone, showing a much higher mortality compared to SG. Therefore, as a first step, we borrowed the life tables estimated by the UN for Sierra Leone in 1950-1980 by assuming that they adequately represented pre-1950 mortality in SG. This allowed us to have estimates of SG mortality going back to quinquennium 1915-1920. Combining the resulting figures of SG age-specific mortality at 1915-1920 with ancient-regime SG fertility led to a (demographic) net reproductive rate NRRin the region of 1.4, therefore still inconsistent with full population stationarity which requires NRR=1. As a next step we therefore supplied a number of simple reconstructions of the Sierra Leone ancien-regime life-table, meant as a life-table which, combined with SG age-specific fertility rates $f_{AR}^{SG}(x_i, n)$, allowed the attainment of a stationary population. The simplest approach was based on a scaling of the $L(x_i, n)$ function by means of a single proportionality factor q (0<q<1) that - once applied to all ages but zero yielded a NRR equal to one. This procedure essentially assumes that all progress in mortality prior to 1915-1920 was concentrated on infant mortality, yielding an unaltered profile of life expectancy at ages different from birth. The resulting female (male) life table implied roughly 50% of each birth cohort eliminated during the first year of life, and 60% before age 5, with a female (male) life expectancy at birth was 20.9 (18.6) yr, while life expectancy at age 5 was about 43 (38).

The SG *ancien-régime* life tables for females and males reconstructed by this approach are reported in Tables S1.2 & S1.3 below.

Age	l(x)	a(x,n)	d(x,n)	L(x,n)	m(x,n)	e(x)
0	100000	0.35	45684	70305	0.649792	20.9
1	54316	1.361	11734	186298	0.062987	37.3
5	42582	2.5	3043	205302	0.014821	43.1
10	39539	2.5	1484	193986	0.007649	41.3
15	38055	2.596822	2009	185450	0.010831	37.8
20	36047	2.534737	2366	174401	0.013566	34.8
25	33681	2.493768	2381	162437	0.014656	32.0
30	31300	2.488727	2298	150730	0.015245	29.3
35	29002	2.483242	2253	139341	0.01617	26.4
40	26749	2.482902	2123	128402	0.016535	23.4
45	24626	2.533617	2074	118015	0.017574	20.2
50	22552	2.575955	2473	106765	0.023164	16.8
55	20079	2.606186	2976	93272	0.031902	13.6
60	17103	2.588506	4022	75817	0.053053	10.5
65	13081	2.522092	4500	54256	0.082932	7.9
70	8582	2.417268	4299	31803	0.135187	5.7
75	4282	2.237731	2892	13422	0.21546	4.1
80	1390	1.967602	1148	3469	0.331083	2.9
85	242	2.125813	242	514	0.470408	2.1

Table S1.2 The reconstructed SG "*ancien-régime*" female life table. Column 2: survivor function l(x), representing the number still alive at each exact age x. Column 3: a(x,n), representing the average number of person-years lived by those dying in each age group (x,x+n). Column 4: d(x,n), representing the number dying in each age group. Column 5: L(x,n), representing the number of person-years lived in each age group. Column 6: m(x,n), representing the mortality rate in each age group. Column 7: e(x), the life expectancy at each exact age x.

	age	l(x)	a(x,n)	d(x,n)	L(x,n)	m(x,n)	e(x)
Sex	0	100000	0.33	50188	66374	0.75614	18.6
Male	1	49812	1.35	13056	164677	0.07928	33.0
Male	5	36756	2.50	2205	178271	0.012366	38.3
Male	10	34552	2.50	1004	170250	0.005895	36.7
Male	15	33548	2.66	1469	164297	0.008943	33.6
Male	20	32079	2.56	2114	155234	0.013621	31.0
Male	25	29965	2.47	1972	144834	0.013619	28.5
Male	30	27992	2.49	1836	135356	0.013564	26.1
Male	35	26156	2.53	1894	126097	0.015018	23.5
Male	40	24262	2.53	2080	116183	0.017904	20.7
Male	45	22182	2.54	2228	105438	0.021127	17.8
Male	50	19955	2.56	2537	93574	0.027109	14.8
Male	55	17418	2.57	2900	80039	0.036228	11.9
Male	60	14518	2.55	3462	64119	0.053998	9.3
Male	65	11056	2.51	3691	46088	0.080081	7.3
Male	70	7365	2.43	3500	27816	0.125811	5.7
Male	75	3866	2.27	2474	12576	0.196749	5.0

Male	80	1392	2.04	1104	3689	0.299217	6.2
Male	85	288	2.27	288	4971	0.057904	20.6

Table S1.3. **The reconstructed SG "*ancien-régime*" male life table**. Column 2: survivor function l(x), representing the number still alive at each exact age x. Column 3: a(x,n), representing the average number of person-years lived by those dying in each age group (x,x+n). Column 4: d(x,n), representing the number dying in each age group. Column 5: L(x,n), representing the number of person-years lived in each age group. Column 6: m(x,n), representing the mortality rate in each age group. Column 7: e(x), the life expectancy at each exact age x.

A summary overview of the evolution of the SG female life tables as depicted by the survivor function is reported in Fig. S1.3 which includes (A) the reconstructed ancien-régime life-table, (B) the intermediate Sierra-Leone life- tables used to estimate SG life tables 1915-1945, (C) the estimated SG life-tables 1950-2015.

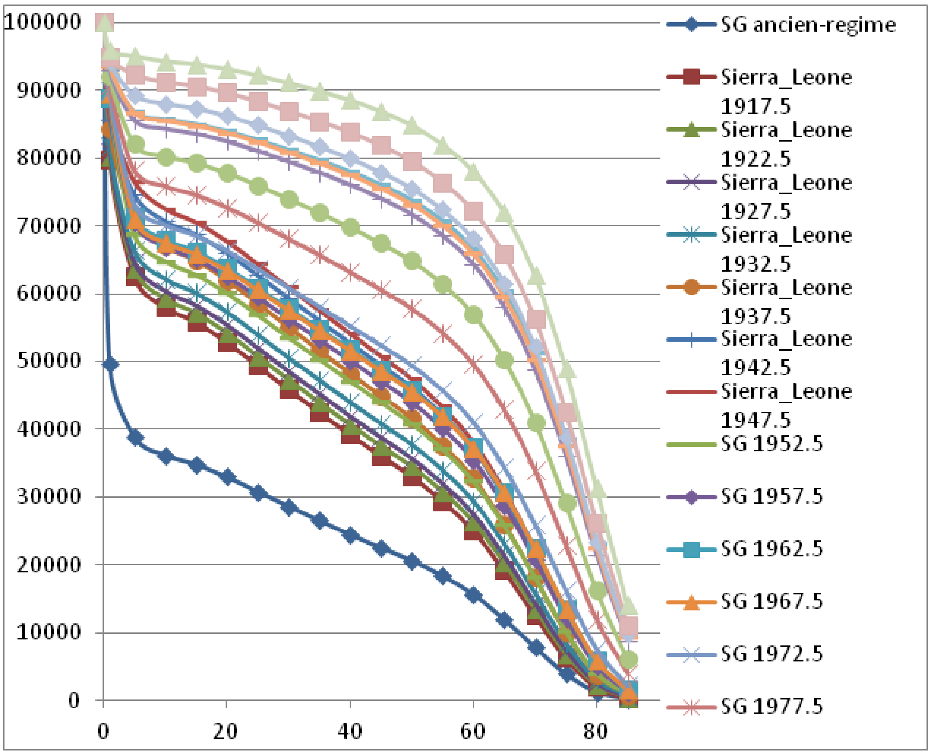

Fig. S1.3. Evolution of SG female survivor function from the *ancien-régime* up to 2015 (based on UN "medium" variant for the period 2015-2100).

Also alternative approaches were used to reconstruct an *ancien-régime* life table for SG, for example by projecting into the past the time series of probabilities of dying at each age estimated by the UN during 1950-1990. Though the results could differ somewhat between themselves, the epidemiological results on the prediction of the course of HBV during the DT reported in the main text are fairly robust with respect to the reconstructed *ancien-régime* life table.

Text S2: Model equations

The age-structured model of population and HBV transmission dynamics is based on the following system of partial differential equations (PDE) and related boundary conditions.

$$\frac{\partial X_g}{\partial a}+\frac{\partial X_g}{\partial t}=dX_g\left(a,t\right)-\left[\lambda_g\left(a,t\right)+\tau\left(a,t\right)+\mu_g\left(a,t\right)\right]X_g\left(a,t\right) \quad (1.1)$$

$$\frac{\partial H_g}{\partial a}+\frac{\partial H_g}{\partial t}=\lambda X_g\left(a,t\right)-\left[\sigma_1+\mu_g\left(a,t\right)\right]H_g\left(a,t\right) \quad (1.2)$$

$$\frac{\partial Y_g}{\partial a}+\frac{\partial Y_g}{\partial t}=\sigma_1 H_g-\left[\sigma_2+\mu_g\left(a,t\right)\right]Y_g \quad (1.3)$$

$$\frac{\partial C_g}{\partial a}+\frac{\partial C_g}{\partial t}=p_c\sigma_2 Y_g\left(a,t\right)-\left[\sigma_3+\mu_g\left(a,t\right)\right]C\left(a,t\right) \quad (1.4)$$

$$\frac{\partial Z_g}{\partial a}+\frac{\partial Z_g}{\partial t}=\left(1-p_c\right)\sigma_2 Y_g\left(a,t\right)+\sigma_3 C_g\left(a,t\right)-\mu_g\left(a,t\right)Z_g \quad (1.5)$$

$$\frac{\partial V_g}{\partial a}+\frac{\partial V_g}{\partial t}=\tau\left(a,t\right)X_g\left(a,t\right)-\mu_g\left(a,t\right)V_g\left(a,t\right) \quad (0.1)$$

where:

$X_g(a,t)$ = number of people of gender *g* and age *a*, who at time *t* are susceptible to HBV infection

$H_g(a,t)$ = number of people of gender *g* and age *a*, who at time *t* have latent HBV infection;

$Y_g(a,t)$ = number of people of gender *g* and age *a*, who at time *t* have acute HBV infection;

$C_g(a,t)$ = number of people of gender *g* and age *a*, who at time *t* have chronic HBV infection;

$Z_g(a,t)$ = number of people of gender *g* and age *a*, who at time *t* are in the recovered state;

$V_g(a,t)$ = number of people of gender *g* and age *a*, who at time *t* are immune to HBV infection thanks to successful immunization;

The population of gender *g* and age *a* at time *t* is given by:

$$N_g(a,t) = X_g(a,t) + H_g(a,t) + Y_g(a,t) + C_g(a,t) + Z_g(a,t) + V_g(a,t) \quad (0.2)$$

In particular the only non-zero boundary conditions are those for the compartments of susceptible individuals and of those suffering perinatal infection

$$X_{0,g}(0,t) = \pi_g \int_{a_3}^{a_4} v(a,t)\left[N_f(a,t) - \left(\theta_Y Y_f(a,t) + \theta_C C_f(a,t)\right)\right] da \quad (1.8)$$

$$H_{0,g}(0,t) = \pi_g \int_{a_3}^{a_4} v(a,t)\left[\theta_Y Y_f(a,t) + \theta_C C_f(a,t)\right] da \quad (1.9)$$

Moreover:

$\mu_g(a,t)$ = background mortality rate *per annum* experienced by those of gender *g* and age *a* at time *t*;

$\lambda_g(a,t)$ = HBV force of infection experienced by people of gender *g* and age *a*, at time *t*;

σ_1 = rate *per annum* of transition from latent to acute HBV infection;

σ_2 = rate *per annum* of recovery/transition from acute to chronic HBV infection;

σ_3 = rate *per annum* of recovery from chronic HBV infection;

$\tau(a,t)$ = proportion effectively vaccinated at age *a* and time *t*;

p_c = age dependent probability of becoming a carrier following acute infection {Edmunds, 1996};

$N_f(a,t)$ = number of women at age *a*, time *t*;

$v(a,t)$ = fertility rate *per annum* at age *a* and time *t*;

(a_3,a_4)= fertile age span

π_g = proportion of births of gender *g*;

θ_Y = proportion of vertically infected births to acutely infected mothers at time *t*;

θ_C = proportion of vertically infected births to chronically infected mothers at time *t*;

Post natal force of infection: This is gender and age-specific and it is defined at each time point t as the sum of the age-dependent (but gender-independent) horizontal force of infection and of the age-gender –specific sexual force of infection (Garnett & Anderson, 1993):

$$\Lambda_g(a,t) = \pi(a,t) + \lambda_g(a,t) \quad (1.10)$$

Horizontal force of infection: This represents the age-specific rate at which susceptible individuals of age a acquire HBV infection through direct ("horizontal") social contacts, per unit of time

$$\pi(a,t) = \frac{\int_{\alpha_1}^{\alpha_2} \beta_1'(a,\alpha)\left[Y_m(\alpha,t)+Y_f(\alpha,t)+\kappa\left(C_m(\alpha,t)+C_f(\alpha,t)\right)\right]d\alpha}{\int_{\alpha_1}^{\alpha_2}(N_m(\alpha,t)+N_f(\alpha,t))d\alpha} \qquad (1.11)$$

where $\beta'(a,\alpha)$ represents the per-capita age-specific transmission rates, and κ the infectiousness of persons chronically infected relative to that of persons with acute infection. We follow the standard assumption that the horizontal force of infection is piecewise constant (Anderson and May 1991) so that the transmission rates $\beta'(a,\alpha)$ can be represented into the form of a WAIFW ("who acquires infection from whom") matrix of elements β_{ij}. We used the following age groups (in completed years) relevant for horizontal transmission: 0, 1-4, 5-9, 10-14, 15+ (Edmunds et al 1996).

Sexual force of infection of HBV: This represents the age-gender specific rate at which susceptible individuals of gender g and age a acquire HBV infection through heterosexual sexual contacts, per unit of time:

$$\lambda_g(a,t) = \int_{\alpha_1}^{\alpha_2} \rho_g(a,\alpha,t)\, c_{g'}(\alpha,t) \frac{\beta_{1g'}Y_{g'}(\alpha,t)+\beta_{2g'}C_{g'}(\alpha,t)}{N_{g'}(\alpha,t)} d\alpha \qquad (1.12)$$

Where

a_1 , a_2 = age at onset and cessation of sexual activity, respectively

$c_{g'}(\alpha,t)$ = average numbers of new sexual partners *per annum* by people of gender g' at age α, time t

$\rho_g(a,\alpha,t)$ = contact or mixing matrix, representing the proportion of sexual partners at time t which people of age a, gender g have with people of the other gender having age α;

$\beta_{1g'}$, $\beta_{2g'}$ = risk of transmission of HBV in a partnership with an infected person of gender g' in, respectively, the stages of acute and chronic infection.

In particular, the mixing matrix is specified according to the so-called preferential rule (Garnett and Anderson 1993), according to which individuals of given gender and age choose their sexual partners in proportions which are an average of the limit cases of perfect age-assortativeness (where all partners are chosen in the same group as own) and of proportionate mixing (where partners are chosen at random):

$$\rho_g(a,a',t) = \varepsilon\delta_{a,a'} + (1-\varepsilon)\mathrm{P}_g(a',t) \qquad (1.13)$$

where

ε is a weighting factor ranging between 0 and 1, determining the degree of assortative sexual mixing by age group (actually here we took ε as age-independent).

$\delta_{a,a'}$: is equal to one for $a = a'$ and zero elsewhere and corresponds to the case of a fully age-assortative mixing matrix, defined as follows (Garnett and Anderson 1993):

$$\delta_{A,A'} = 1, \quad A = A'$$
$$\delta_{A,A'} = 0, \quad A \neq A' \qquad (1.14)$$

$P_g(a',t)$ represents a proportionate (or random) sexual mixing matrix, defined as follows (Garnett and Anderson 1993)

$$P_g(a,t) = \frac{c_{g'}(a,t)N_{g'}(a,t)}{\int_0^\infty c_{g'}(\alpha,t)N_{g'}(\alpha,t)d\alpha} \qquad (1.15)$$

In particular ages a, a' relevant for sexual transmission are discretized into 5 year age bands (15-19, 20-24, etc)

Balancing sexual partner numbers

To ensure sexual partner numbers balance despite possible relative changes in numbers in each subgroup during the dynamics of the system there are a number of possible solutions (Garnet & Anderson 1993); here we used the following rules

$$c'_m(a,t) = c_m(a,t)\left[1 - \frac{\int_0^\infty c_m(a,t)N_m(a,t) - c_f(a,t)N_f(a,t)}{\int_0^\infty c_m(a,t)N_m(a,t) + c_f(a,t)N_f(a,t)}\right]$$
$$c'_f(a,t) = c_f(a,t)\left[1 + \frac{\int_0^\infty c_m(a,t)N_m(a,t) - c_f(a,t)N_f(a,t)}{\int_0^\infty c_m(a,t)N_m(a,t) + c_f(a,t)N_f(a,t)}\right] \qquad (1.16)$$

Age-distributed sexual contact rates

Baseline sexual contact rates $\kappa_g(a,t)$ were scaled by a factor $\hat{\Theta}_A$ triangular distributed according to five-yearly age group, A, and used to calculate $c_g(a,t)$:

$$\hat{\Theta}_A = \theta' + \frac{(A - A_{\min-1})}{(\mathrm{M} - A_{\min-1})H}, \quad A \leq \mathrm{M}$$
$$\hat{\Theta}_A = \theta' + \frac{(A_{\max+1} - A)}{(A_{\max+1} - \mathrm{M})H}, \quad A > \mathrm{M}$$
$$H = \theta' \frac{\sum_{A_{\min}}^{M} \frac{(A - A_{\min-1})}{(\mathrm{M} - A_{\min-1})} + \sum_{M+1}^{A_{\max}} \frac{(A_{\max+1} - A)}{(A_{\max+1} - \mathrm{M})}}{A_{\max} - A_{\min-1}} \qquad (1.17)$$
$$c_g(a,t) = \hat{\Theta}_A \kappa_g(a,t)$$

with Latin hypercube sampled parameters mode, M, corresponding to age group, and baseline value, θ'.

Table S2.1: Model parameters. Ranges (uniformly distributed) of parameter values used in the modelling used to generate model predictions.

S2.1a Latin hypercube sampled parameters.

Parameter	Units	Prior range		Best fit value	Source
		Lower limit	Upper limit		
Duration of latent infection	months	1.5	3.0	2.91	*
Duration of acute infection	months	2.25	6.0	2.71	*
Duration of persistent infection	years	40	60	41.8	*
Female risk of infection following unprotected sexual partnership with acutely infected male	/partner	0.425	0.69	0.465	*
Risk of female to male sexual transmission relative to male to female risk	-	0.225	0.925	0.284	‡
Risk of sexual or horizontal transmission from someone with chronic infection relative to risk from someone with acute infection	-	0.120	0.1875	0.151	*
Risk of vertical transmission from mother with acute infection to unvaccinated newborn	-	0.475	0.875	0.613	*
Risk of vertical transmission from mother with chronic infection to unvaccinated newborn	-	0.185	0.4	0.220	**
Scaling factor for WAIFW matrix	-	0.775	1.125	1.05	‡
Relative weighting of adopted WAIFW ††	-	0.725	1.0	0.933	‡
Degree of age-related assortativeness of sexual contacts‡	-	0.225	0.975	0.784	***
Sexual partners, male	partners /annum	1.75	2.9	2.88	*
Sexual partners, female	partners /annum	1.75	2.9	2.07	*
Baseline value for age-distributed sexual contacts, male	partners /annum	0.3	1	0.404	‡
Baseline value for age-distributed sexual contacts, female	partners /annum	0.3	0.87	0.382	‡
Mode of age-distributed sexual contacts, male †	-	4	8	4.29	‡
Mode of age-distributed sexual contacts, female †	-	4	8	4.25	‡
Duration of transition between *ancien-régime* and intermediate mortality rates	years	8	40	33.1	‡
Duration of transition between intermediate and SG mortality rates	years	30	60	18.9	‡
Duration of period from onset of SG mortality rates to 1950	years			41.6	‡

†NB Value of e.g. 4 for mode corresponds to the 15-19 age group and 8 to the 35-39 age group

†† Weighting of the model A & B matrices of Edmunds *et al*, 1996

‡Assumption

*Range of values around point estimate of Edmunds et al, 1996

** Shepard et al & Howells et al

*** Williams et al, 2014

S2.1b Other parameters with constant value

Parameter	Value	Reference
Acute infections resulting in fulminant disease and death	0.83%	Mina et al
Male:female sex ratio at birth	0.512	Preston et al

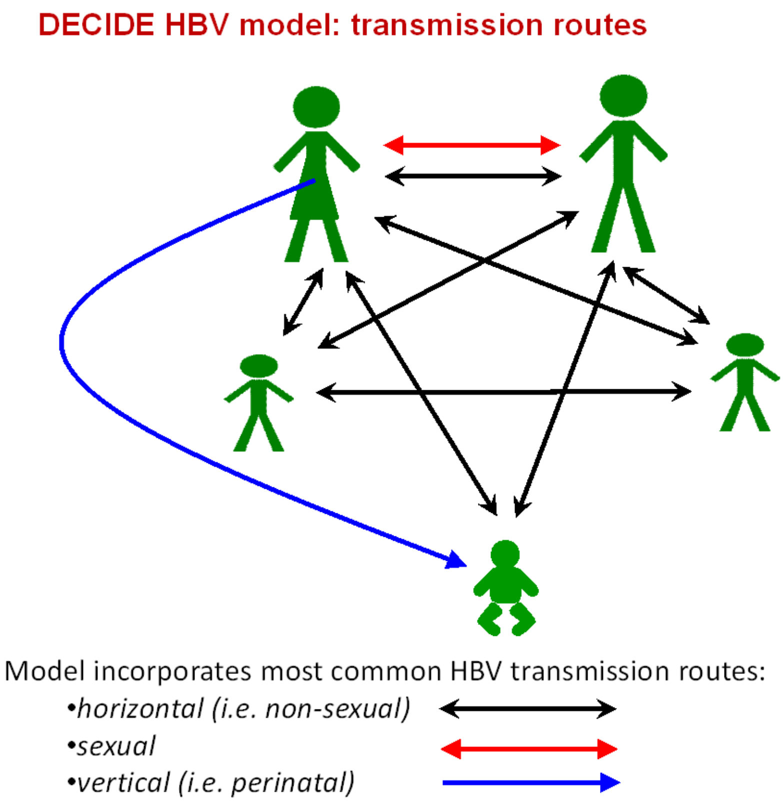

Figure S2.1 HBV transmission routes for adults, children 0-15 years and new-borns incorporated in the model;

Text S3: Model results: uncertainty

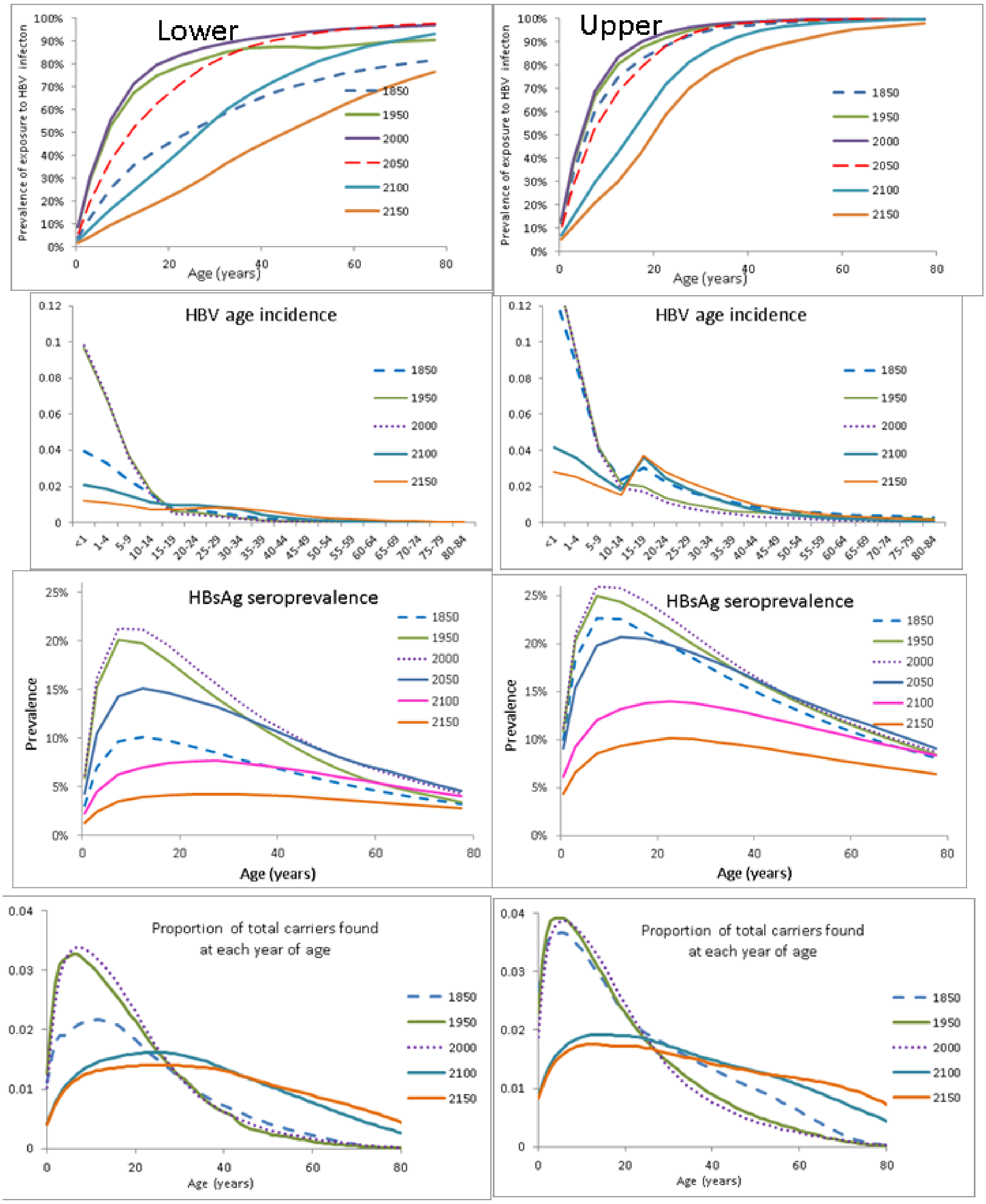

Figure S3.1. Lower (left hand panels) and upper (right hand panels) uncertainty bounds for the predicted evolution of HBV prevalence & incidence during the course of the DT (corresponds to Figure 4 in the main text): (a & b) age-specific HBV prevalence; (c & d) age-specific HBV incidence; (e & f) age-specific prevalence of chronic infection; (g & h) distribution of cases of chronic infection by age.

To assess levels of uncertainty in the results, a series of model runs was carried out using the best 10% of the parameter constellations sampled with LHS, i.e. the 10% giving rise to the lowest values of the least squares function when fitting the HBV data, In analysing these results the maximum and minimum values of the appropriate model outputs at each time point were selected as the upper and lower uncertainty bounds at that time point (see Figures S3.1-S3.3); it is important to note therefore that in each figure the successive points on the upper and lower uncertainty bounds do not necessarily correspond to a single trajectory.

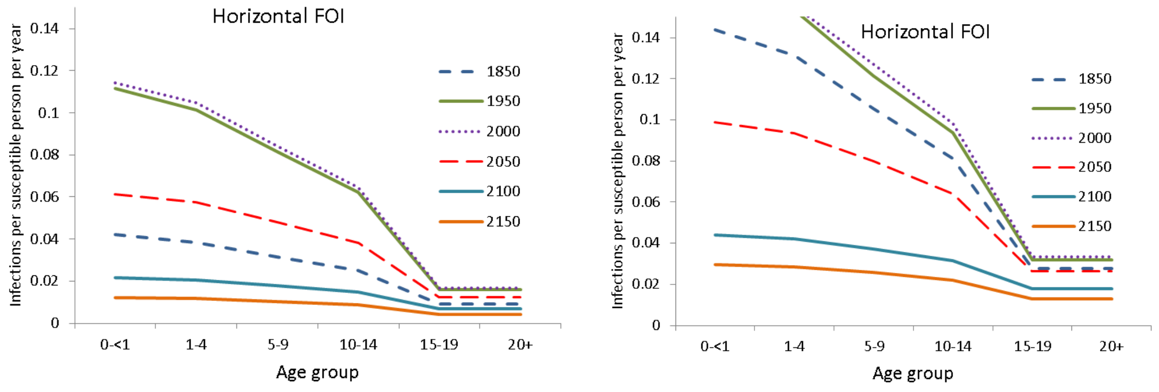

Figure S3.2. Lower (left hand panel) and upper (right hand panel) uncertainty bounds for for the predicted evolution of HBV horizontal force of infection over the course of the DT (corresponds to Figure 5a in the main text).

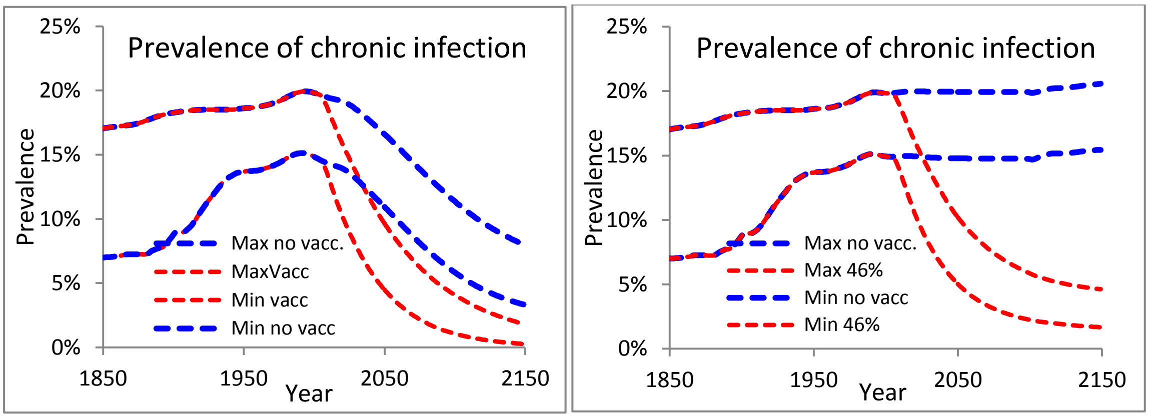

Figure S3.3 Left hand panel shows upper and lower uncertainty bounds for the predicted evolution of prevalence of chronic HBV over the course of the DT; right hand panel shows the upper and lower uncertainty bounds for the counterfactual scenario in which fertility remains constant at its 1990 levels (corresponds to Figure 6 in the main text). In the base case, vaccination is administered at age 3.5 months with coverage of 46% [33] and for comparison results are also shown corresponding to an alternative vaccination programme with coverage of 95%; and assuming in both cases 100% efficacy

Text S4 Vaccination at birth

Lack of availability of monovalent HBV vaccine and issues relating to infrastructure and the logistics of delivering birth doses of vaccine remain substantial barriers to the implementation in the region of effective programmes of vaccination at or shortly after birth, even though this has proved to be an effective tool for the prevention of perinatal HBV infection. Nevertheless, in order to assess the potential impact of a programme of vaccination at birth the modelling also investigated the effectiveness of vaccination at birth in place of infant vaccination but with the same baseline coverage of 46% (Figure S4.1). In the case using the UN medium projections vaccination at birth provides a small but significant advantage compared with infant vaccination, achieving similar levels of reduction in prevalence of chronic infection a decade or two earlier after 50 years or so (Figure S4.1 upper panel). However in the worst case scenario of fertility remaining at 1990 levels the difference is much more marked and while by 2150 infection nears elimination with vaccination at birth, infant vaccination achieves no more by 20150 than prevalence beginning to stabilises at just over 3% (Figure S4.1 lower panel)

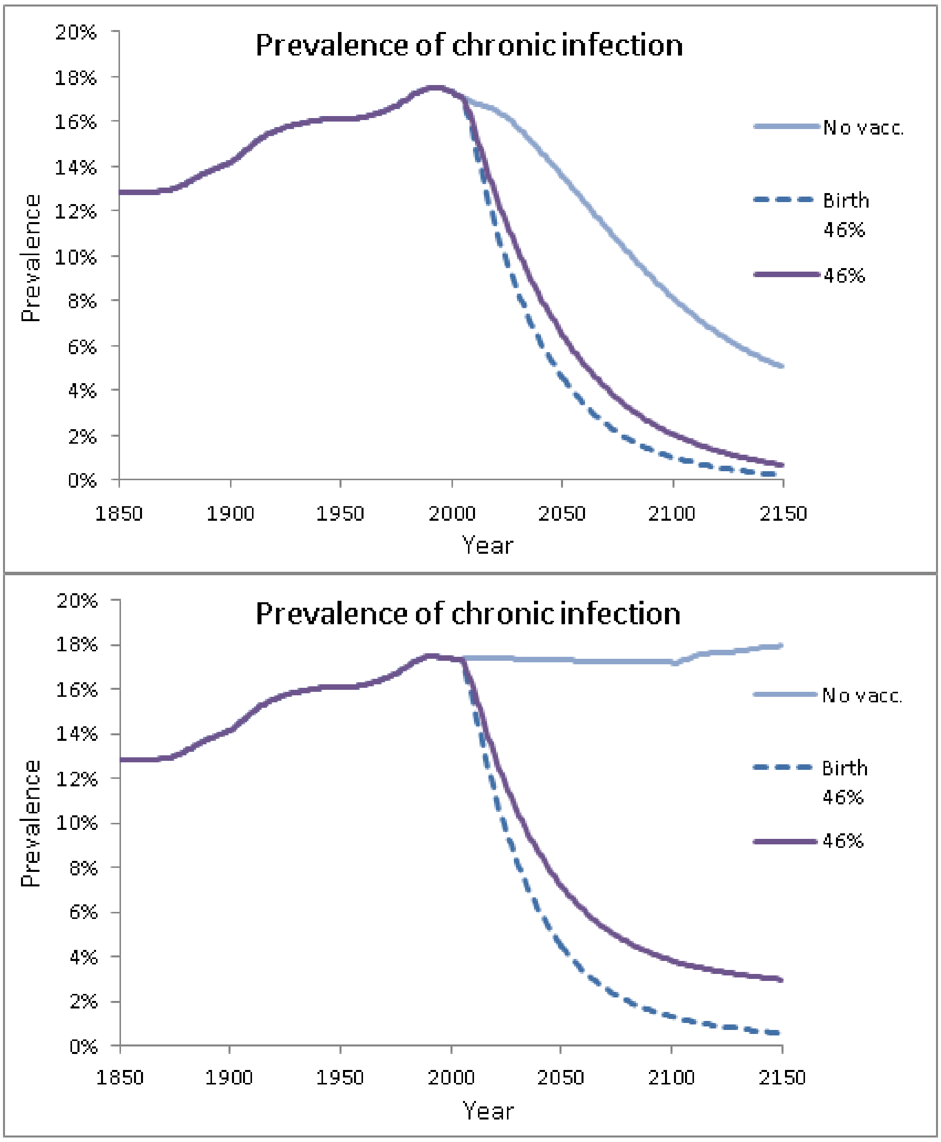

Figure S4.1 HBV disease burden over the DT comparing impact of vaccination at 3.5 months of age with vaccination at birth each with effective coverage of 46% starting in 2005

Text S5 Incidence ratios

Figure 5 in the main text shows model results for temporal change in predicted HBV incidence by transmission route over the entire course of the DT under the UN medium variant. Figure S5.1 shows the ratios between incidence results for horizontal and vertical and horizontal and sexual transmission in order to clarify the relationships between incidence arising via these transmission routes

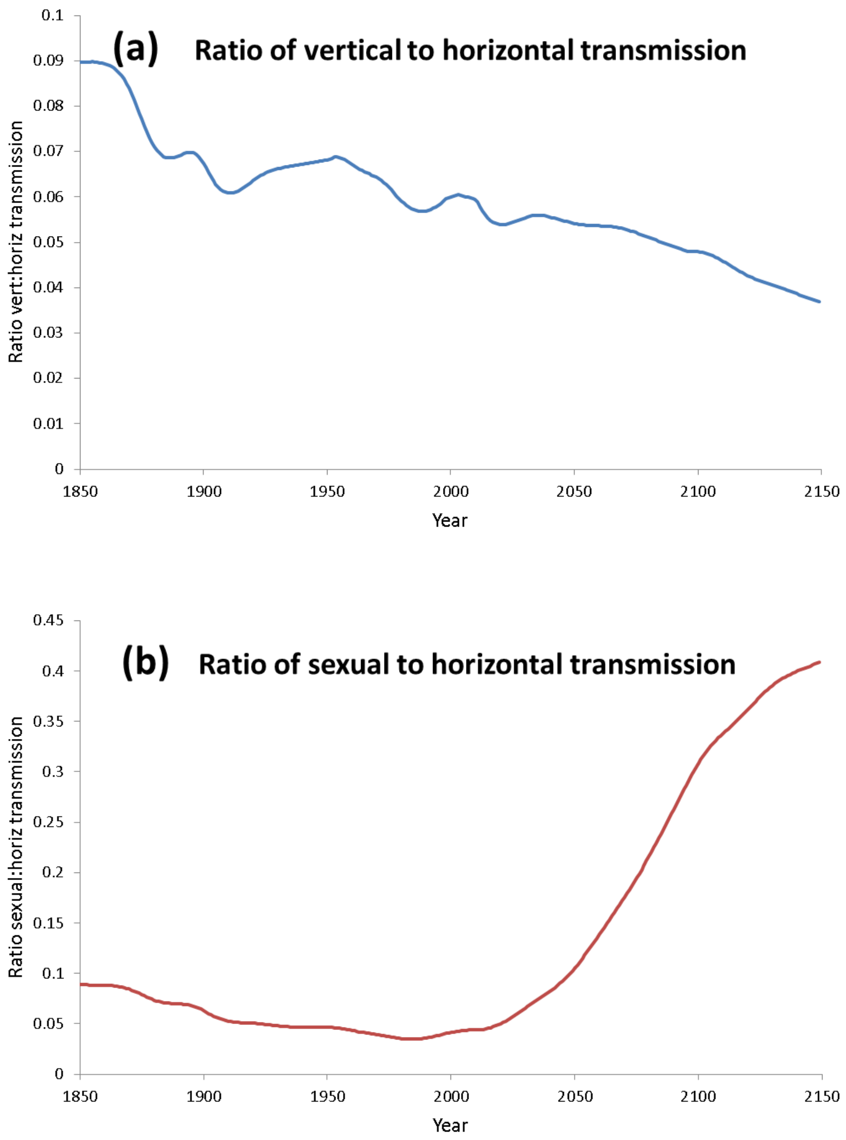

Figure S5.1 Ratio relative to incidence by horizontal transmission of (a) incidence by vertical and (b) incidence by sexual transmission (corresponds to ratios of results shown in Figure 5 in the main text for incidence arising from horizontal, vertical and sexual tranmission).

Text S6 Influence of individual parameters

For each of the model parameters in turn shown in Table S6.1 model runs were undertaken using the values of this parameter found in the 2,400 parameter sets (10% of the total) resulting in the lowest least squares values; at the same time the remaining parameter values were kept at the single best fit value. For each set of model runs varying a single parameter least squares values were calculated for the fit to the Gambian HBsAg seroprevalence data. The distribution of resulting least squares values for each parameters are reported in Figure S6,1 which shows that the parameters with most influence on the fit to HBsAg data were the (i) risk of transmission from someone with chronic infection relative to transmission from someone with acute infection (L), (ii) risk of vertical transmission from mother with chronic infection to unvaccinated newborn (O), and (iii) the scaling factor for the WAIFW matrix (P). Less influential were the durations of latent (A), acute (B) and chronic (C) infection; parameters relating to sexual transmission were the least influential

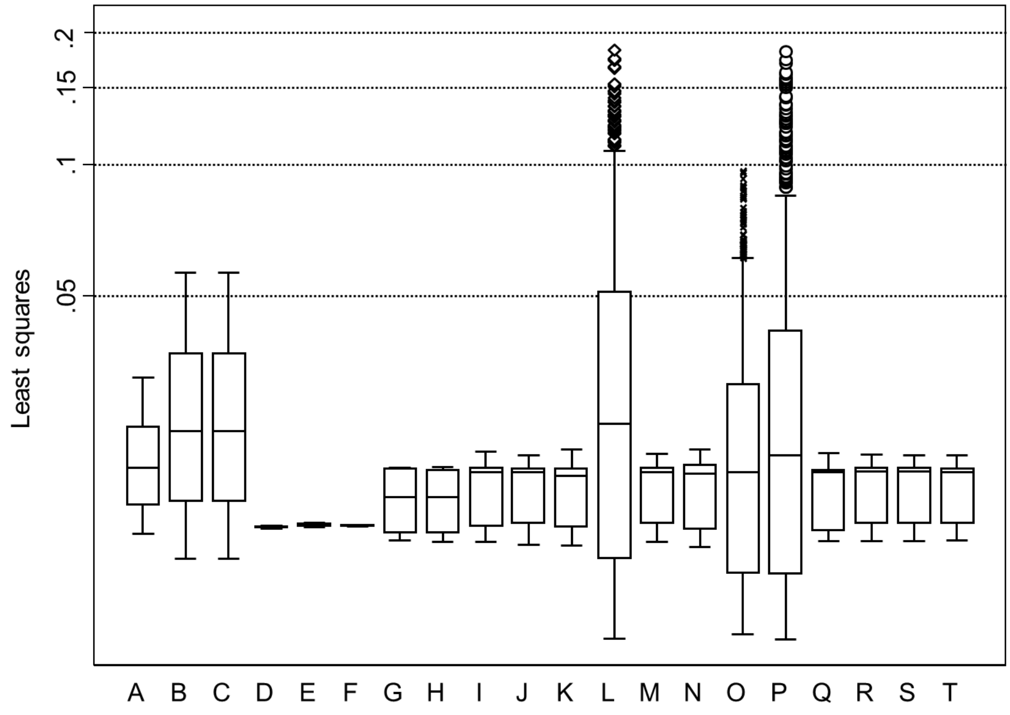

Figure S6.1 Box plots of distribution of least squares values for HBV model parameters. Note that a logarithimic scale is used for the vertical axis (for key see Table S6.1)

	Parameter
A	Duration of latent infection
B	Duration of acute infection
C	Duration of persistent infection
D	Sexual partners per annum, male
E	Sexual partners per annum, female
F	Baseline value for age-distributed sexual contacts, male
G	Baseline value for age-distributed sexual contacts, female

H	Mode of age-distributed sexual contacts, male
I	Mode of age-distributed sexual contacts, female
J	Risk of female to male sexual transmission relative to male to female risk
K	Female risk of infection following unprotected sexual partnership with acutely infected male
L	Risk of sexual or horizontal transmission from someone with chronic infection relative to risk from someone with acute infection
M	Degree of age-related assortativeness of sexual contacts
N	Risk of vertical transmission from mother with acute infection to unvaccinated newborn
O	Risk of vertical transmission from mother with chronic infection to unvaccinated newborn
P	Scaling factor for WAIFW matrix
Q	Relative weighting of WAIFW matrix A vs matrix B
R	Duration of transition between ancien-régime and intermediate mortality rates
S	Duration of transition between intermediate and SG mortality rates
T	Duration of period from onset of SG mortality rates to 1950

Table S6.1 Key to box plot Fig S6.1

References

Adetunji J, Bos ER. **Levels and Trends in Mortality in Sub-Saharan Africa: An Overview. In:** *Disease and Mortality in Sub-Saharan Africa.* Edited by Jamison RG et al. Washington DC: The World Bank Press; 2006.

Anderson RM, May RM: **Infectious diseases of humans: dynamics and control**. Oxford: OUP; 1991.

Dyson T, Murphy M. **The onset of fertility transition.** *Population and Development Review*. 1985:399-440.

Edmunds WJ, Medley GF, Nokes DJ, Hall AJ, Whittle HC: **The influence of age on the development of the hepatitis B carrier state**. *Proc R Soc Lond B* 1993, **253**(1337):197-220.

Edmunds WJ, Medley GF, Nokes DJ: **The transmission dynamics and control of hepatitis B virus in The Gambia**. *Stat Med* 1996, **15**:2215-2233.

Garnett GP, Anderson RM: **Factors controlling the spread of HIV in heterosexual communities in developing countries: patterns of mixing between different age and sexual activity classes.** *Philos Trans R Soc Lond B.* 1993, **342**(1300):137-59.

Howell J, Lemoine M, Thursz M. **Prevention of materno-foetal transmission of hepatitis B in sub-Saharan Africa: the evidence, current practice and future challenges.** *J Viral Hepat.* 2014, **21**(6):381-96.

Keyfitz N, Caswell H: **Applied mathematical demography.** New York: Springer Verlag; 2008.

Mina T, Amini Bavil Olyaee S, Tacke F, Maes P, Van Ranst M, Pourkarim MR. **Genomic Diversity of Hepatitis B Virus Infection Associated With Fulminant Hepatitis B Development.** *Hepat Mon.* 2015, **15**(6):e29477.

Preston P, Heuveline P, Guillot M: **Demography: Measuring and Modeling Population Processes.** New York: Wiley & Sons; 2000.

Shepard CW, Simard EP, Lyn Finelli, Fiore AE, Bell BP. **Hepatitis B Virus Infection:** Epidemiology and Vaccination. *Epidemiol Rev.* 2006, 28:112–125.

Williams JR, Alary M, Lowndes CM, Béhanzin L, Labbé AC, Anagonou S, et al. **Positive impact of increases in condom use among female sex workers and clients in a medium HIV prevalence epidemic: modelling results from Project SIDA1/2/3 in Cotonou, Benin.** *PLoS One.* 2014, 9(7):e102643.
